# Simulation of group testing scenarios can boost COVID-19 screening power

**DOI:** 10.1038/s41598-022-14626-8

**Published:** 2022-07-13

**Authors:** Vinicius Henrique da Silva, Carolina Purcell Goes, Priscila Anchieta Trevisoli, Raquel Lello, Luan Gaspar Clemente, Talita Bonato de Almeida, Juliana Petrini, Luiz Lehmann Coutinho

**Affiliations:** 1 Department of Animal Science, Luiz de Queiroz College of Agriculture (ESALQ), University of São Paulo (USP), Piracicaba, Brazil; 2 Anhembi Morumbi University, Piracicaba, Brazil

**Keywords:** Computational biology and bioinformatics, Microbiology

## Abstract

The COVID-19 has severely affected economies and health systems around the world. Mass testing could work as a powerful alternative to restrain disease dissemination, but the shortage of reagents is a limiting factor. A solution to optimize test usage relies on ‘grouping’ or ‘pooling’ strategies, which combine a set of individuals in a single reaction. To compare different group testing configurations, we developed the *poolingr* package, which performs an innovative hybrid in silico/in vitro approach to search for optimal testing configurations. We used 6759 viral load values, observed in 2389 positive individuals, to simulate a wide range of scenarios. We found that larger groups (>100) framed into multi-stage setups (up to six stages) could largely boost the power to detect spreaders. Although the boost was dependent on the disease prevalence, our method could point to cheaper grouping schemes to better mitigate COVID-19 dissemination through identification and quarantine recommendation for positive individuals.

## Introduction

The SARS-CoV-2 is a virus with crown-like spikes on its surface^1^ and for that reason is classified as a member of the Coronaviridae family^2^. It was initially reported at the end of 2019 in China (Wuhan, province of Hubei), but after a few months it spread globally^3^. The rapid spread of SARS-CoV-2 led to a sharp world-wide increase in the number of COVID-19 cases and the global death toll has surpassed six million^4^. In such a rapidly spreading pandemic, it is challenging to delineate mitigation strategies, which intersect a range of different solutions, players and stakeholders^5^. Social distance measures, development of vaccines and testing programs to isolate spreaders can be pointed as the main strategies of mitigation. Social restrictions, such as lockdown, have been applied to bend the infection curve, but harsh effects on the world economy can be expected^6^. Several vaccines have been developed^7^, which have been applied to generate immunity and therefore halt disease dissemination. However, vaccines can take a long time to generate global immunity and may be less efficient for new viral variants such as the variant Delta or Omicron, which are of special concern as they can escape to antibody neutralization^8,9^. The last strategy, i.e. a testing program, is able to identify possible disease spreaders and then inform these positive individuals about their condition. As a consequence, the number of spreaders in quarantine is expected to increase, which will invariably lead to a decrease in the basic reproduction ratio (${R}_{0}$, reviewed by^10^) in a population.

Testing programs^11^ can focus on the most problematic regions, companies^12^ or social groups^13^, allowing tracking of infected people and therefore reducing disease dissemination. This can be of special importance in the COVID-19 pandemic given that asymptomatic individuals have even a higher viral load than symptomatic COVID-19 patients^14^ and could become superspreaders^15^. Massive testing programs for COVID-19 were implemented around the world^16^, but the scarcity of reagents is a limiting factor to perform many tests. A possible solution to mitigate this problem is the implementation of strategies of group testing to achieve a higher number of results with the same amount of reagents^17^. Group testing relies on the assumption that for groups in which all members are negative just one test is needed to declare all individuals as negative^18^. There are different ‘group’ or ‘pool’ strategies^19^, which can be generally classified as hierarchical^20^ or array^21^. However, although different strategies for group testing exist, all laboratories are ultimately interested in a cost-effective one. To search for the optimum testing configuration (OTC), specific objective functions have been developed.

Objective functions aim the OTC^22,23^, which depends on disease prevalence, assay sensitivity and/or specificity as well as the group size range allowed in a laboratory setup. Although most of these objective functions are fast and reliable under certain assumptions, the group size effect on assay sensitivity, i.e. the dilution effect^24^, is usually considered constant among group sizes^25^ and therefore do not precisely estimate the general sensitivity in a specific OTC. Otherwise, the user is recommended to place an upper limit to define the maximum group size allowed for an OTC and manually define a general sensitivity^26^. Therefore, these functions may consider the same sensitivity for all group sizes allowed by a predetermined range. Moreover, multi-staged OTCs can have just up to three stages in widely-used shiny applications such as *PooledTesting*^23,26–31^, although up to six stages can be explored by the *binGroup2*^32^ R package and in a multiplex-group testing strategy^30^. It is important for a testing program to consider multi-stage testing configurations as it has been reported to outperform several other approaches in the literature^33^. Last, the metric that is usually applied to rank testing configurations may be unsuitable for the COVID-19 pandemic. The *number of tests per result* is useful to see how many tests are needed to achieve a certain number of individual results. However, to use such a metric, it is necessary to set a minimum sensitivity to avoid configurations that have too high dilution effect (i.e. low sensitivity). Moreover, to mitigate COVID-19 dissemination, an increase in power to identify positive individuals, i.e. potential spreaders, may be more relevant.

We propose a metric to rank testing configurations, named as *boost in spreader detection*, which is represented by the ratio between the number of possible spreaders (i.e. positive individuals) identified by a testing configuration in comparison with individual testing. In such way, it is possible to understand how powerful a testing configuration is to pinpoint spreaders, whereas the sensitivity decrease caused by grouping dilution effect is considered quantitatively. To evaluate testing configurations by their *boost in spreader detection* we developed a comprehensible and open-source R package, called *poolingr*, designed to infer OTCs by simulating a range of configurations. The initial assumptions include disease prevalence, assay sensitivity and specificity as well as a list of viral loads values from RT-qPCR tests (i.e. cycle threshold, Ct) that were observed in positive individuals. In these simulations, Ct values are randomly selected as simulated scenarios require it. Therefore, the dilution effect of group size and disease prevalence can be jointly considered. We explored different scenarios under a range of testing configurations using the Ct values from more than two thousand individuals tested as positive for SARS-CoV-2. Multi-stage testing configurations are useful to increase the power to screen spreaders and *in silico* simulations can be useful to quantify the boost of a specific testing configuration.

## Results

### Group size and disease prevalence exert a complementary dilution effect

**Figure 1:**
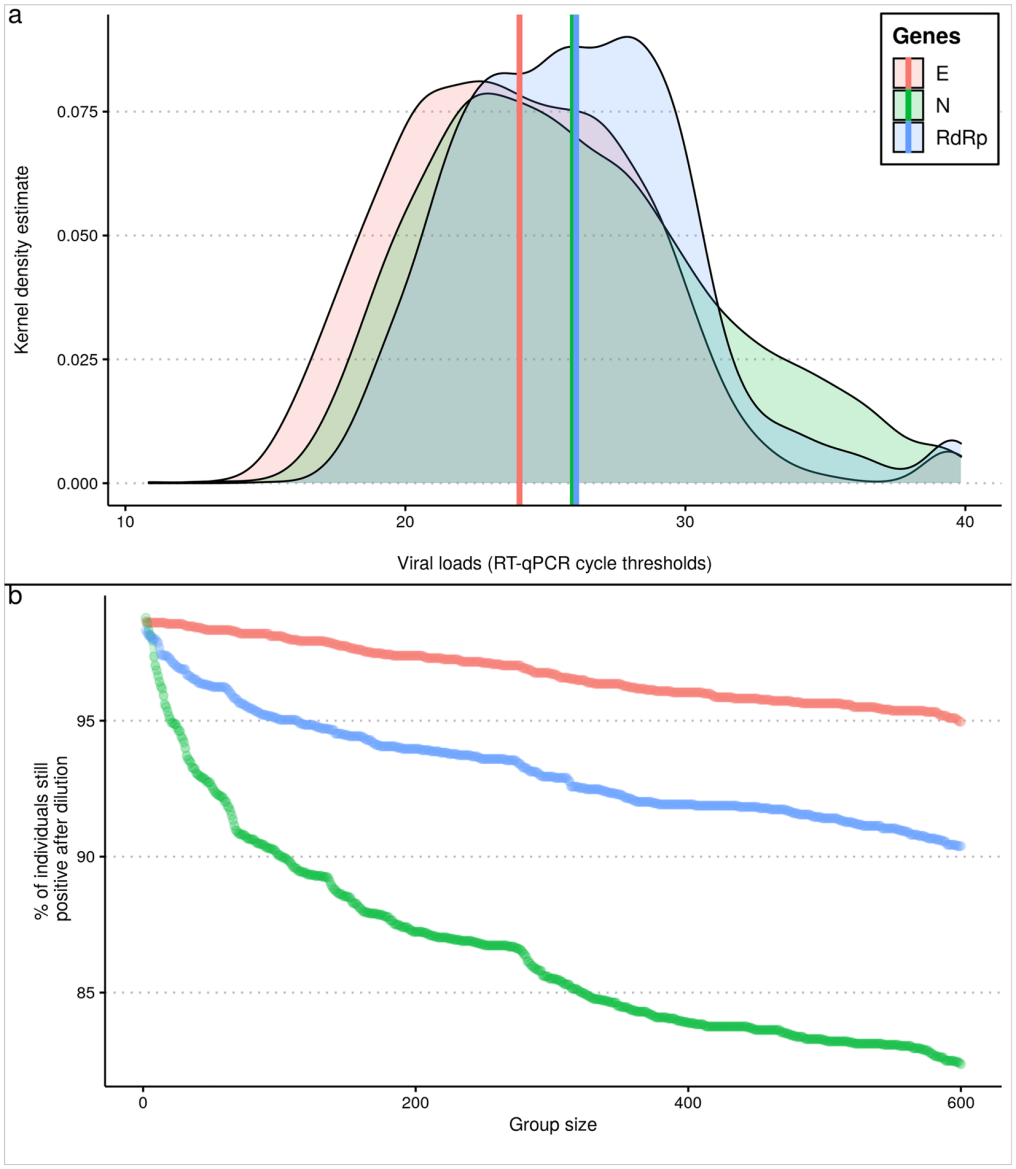
Expected dilution effect in observed SARS-CoV-2 viral loads. (**a**) Distribution of the SARS-CoV-2 viral load (i.e. RT-qPCR cycle thresholds) values among 2,389 positive individuals. The vertical lines indicate mean Ct values for each gene. These mean values were not used by the simulations as the Ct values were randomly selected as given scenarios required it. (**b**) Proportion of the analyzed positive individuals that in theory would still retrieve a positive result in groups from 2 to 600 for which the groups considered only one positive sample with negative samples (i.e. individual sample after dilution).

Mixing positive and negative individual samples in one reaction will dilute the amount of genetic material of the pathogen that is under test. Therefore, it is important to quantify such dilution effect properly before implementing a strategy for group testing. In this report, we used in vitro quantification values from individual positive COVID-19 tests obtained from an outbreak investigation in Brazil. We used a total of 2,389 positive individuals analyzed for three different probe-sets targeting E (Envelope protein), N (Nucleocapsid) and RdRp (coding region of Orf1ab polyprotein) viral genes, with total of 6,759 RT-qPCR Ct values. These values ranged from 10.83 in E gene to 39.84 in RdRp gene among all individuals. The average Ct value was 24.08, 25.98, and 26.1 in E, N, and RdRp , respectively (Fig. 1a).

Groups with positive individuals are expected to be positive, at least before the maximum dilution (based on the viral load and group size) is reached, permitting the detection of viral fragments by the PCR machine. Thus, samples with relatively higher Ct values may become undetectable more frequently, mainly when large groups are applied. Here we considered as positive all individuals (i.e. in vitro) or groups (i.e. in silico) with a RT-qPCR Ct value below 40. The Ct of different group sizes with different combinations of positive individuals can be predicted in silico due to the log-linear dilution effect expected by grouping samples analyzed by quantitative PCR (qPCR). The theoretical Ct value of a group can be estimated by the formula ${\log _a}(group.size)+ct.individual$, where *a* is equal to $1 + pcr.efficiency$. For convenience, this formula was implemented in the CtInPool function of the *poolingr* package. In theory, the great majority of the individuals could be detectable in very large groups (>100, Fig. 1b). This is expected because all samples with a Ct values up to $\approx$30.77 would still be detectable when diluted with other 599 negative samples, given that the group Ct would be $\approx$39.99 (i.e. below 40).

The estimation of dilution effect for an actual testing program, in a real population, may need to consider how the positive individuals will be distributed across the qPCR reactions (group or individual tests) under an assumed disease prevalence. Thus, to estimate more precisely the dilution caused by group sizes ranging from two to 600 individuals, we used our *poolingr* package to simulate ten iterations for a population with ten thousand individuals, under seven scenarios varying from 0.1 to 30% of prevalence. Each iteration generated a binary index to represents the simulated population, where positive individuals were randomly placed. Chunks of the index, for which the length is equal to the simulated group size, were used to estimate the group viral load (i.e. group Ct) with different random combinations between negative and positive individuals. As more than one positive individual can be present in a group, a two-step procedure with the uniroot function adapted from CtInPool function was implemented in the simulations (see methods and source-code for details). Considering as positive only groups with Ct below 40, for N or RdRp viral regions, the sensitivity was defined as the proportion between groups considered positive and those containing at least one positive individual in the simulation. As E gene characterizes the subgenus *Sarbecovirus* and N and RdRp were specific to SARS-CoV-2, we considered as positive the individuals presenting Ct for at least one of the two specific genes (N or RdRp).

**Figure 2:**
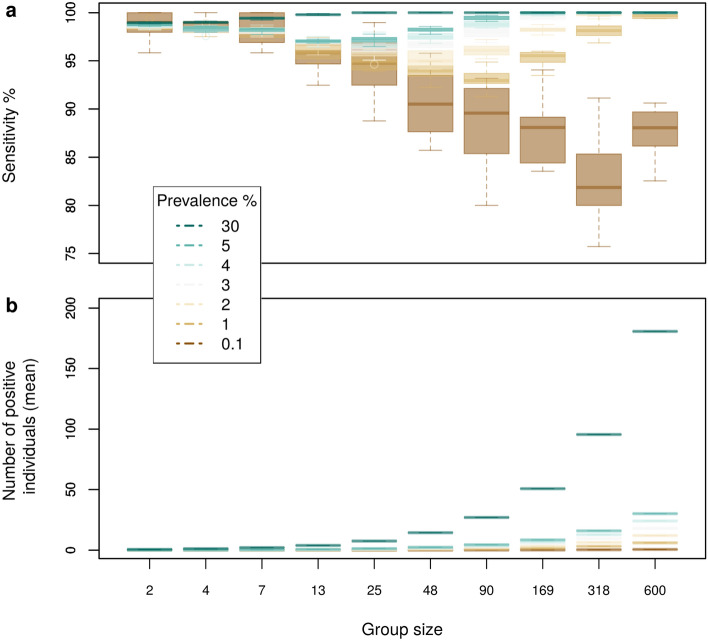
Effect of group size and prevalence on test sensitivity. Simulations were performed for seven prevalence scenarios and ten group sizes with 100 simulation iterations each. (**a**) The sensitivity of the test tends to decrease as the group size increases and this effect is greater, and has greater variability, at the lower prevalence tested. (**b**) In the higher prevalence tested, the sensitivity in larger groups is still high due to the likelihood that they contain relatively more positive individuals. In this case, the dilution effect is mitigated by disease prevalence.

The dilution effect was translated into group sensitivity (i.e. percent of the positive group chunks that are detectable after dilution in each group size iteration), which was close to 100% in small groups but plunged down to $\approx$75% in at least one simulated iteration considering groups with 318 individuals, considering the minimum prevalence of 0.1% . However, such a dilution effect was mitigated as prevalence increased (Fig. 2a). At the lower prevalence tested, most of the simulated groups are negative and positive groups usually contain only one individual that is positive. Although it is rare to have more than one positive individual in a group when the prevalence is low, as it increases, a higher proportion of the groups will be positive or/and contain more than one positive individual just by chance. This is reflected by a convex curve formed by the relationship between group size and sensitivity over crescent prevalence values (Fig. 2b).

### Dilution effect should be considered to identify more spreaders with less resources

Defining a minimum sensitivity threshold to filter out undesirable testing configurations is not a trivial issue when screening for potential spreaders. If no group testing is implemented for a population, one RT-qPCR reaction per individual would be needed to give a result to everyone. If we assume disease prevalence of 0.1% in the population, a testing program would spend 1,000 reactions per detected spreader. To understand how a testing configuration can improve resource usage, we propose a comparative metric called *boost in spreader detection*, which represents the ratio of the reactions per detected spreader between a (1) given testing configuration and (2) individual testing. Thus, this metric directly depends on the general sensitivity of the configuration (i.e. weight of the dilution effect on the applied group sizes) and may reflect it quantitatively. Ct values observed in vitro were used for in silico simulated testing configurations using our *poolingr* package. The simulations were based on a parallelized brute-force evaluation of a wide range of group testing scenarios with randomly selected individuals. In this section, we used the observed viral loads in our *poolingr* package to estimate this boost by using testing configurations that have a group test followed by individual testing (i.e. two-stage configuration) and in the next section we explore more complex multi-stage configurations using the same metric.

**Figure 3:**
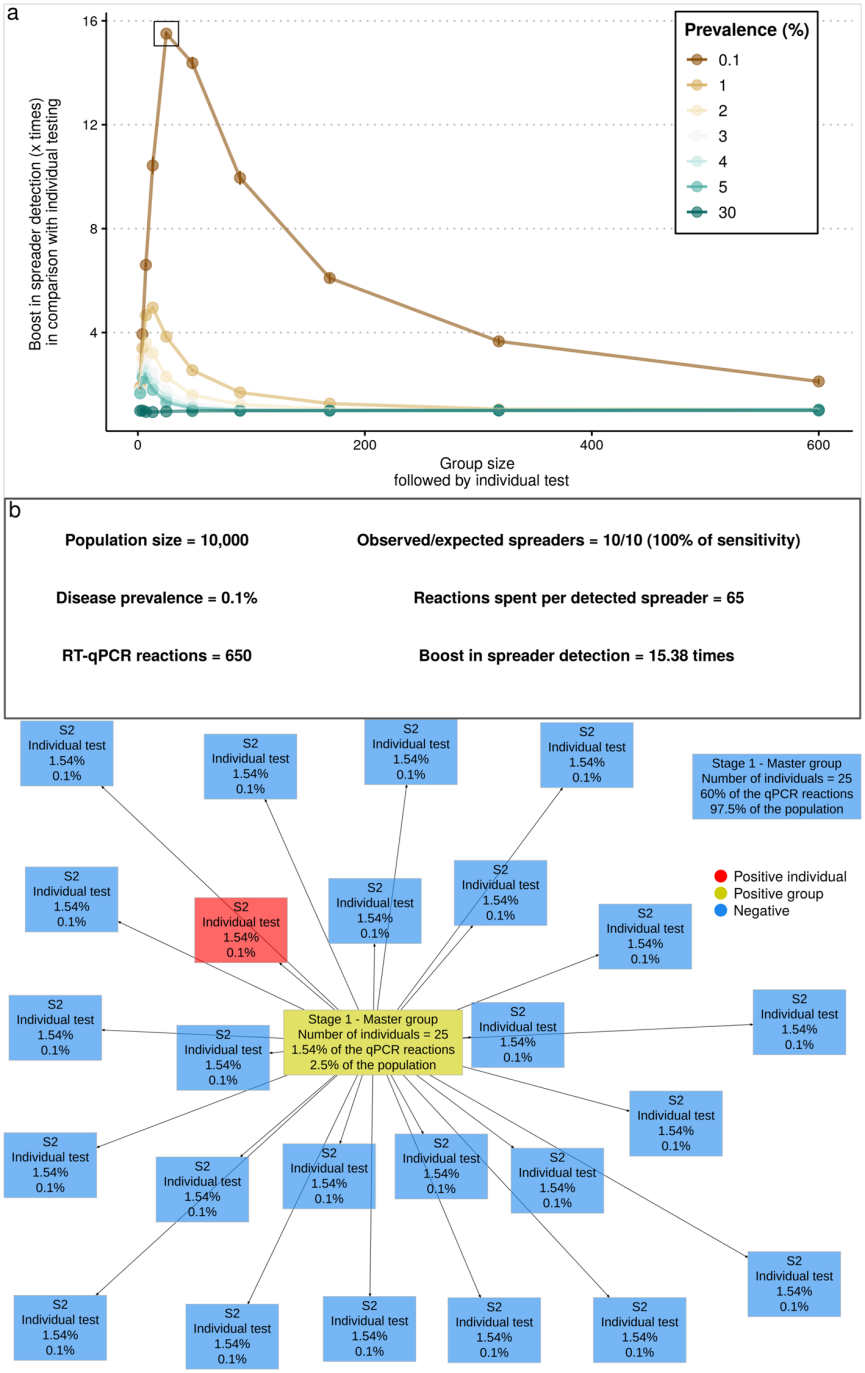
Boost in spreader detection as group size increases in double-stage testing configurations (TCs). Double-stage TCs represent group tests that are followed by an individual test. (**a**) Simulation of ten thousand individuals with disease prevalence ranging from 0.1 to 30% . The *x*-axis shows all simulated group sizes and the *y*-axis the boost of the respective TC in the screening power to detect COVID-19 spreaders. The square is around the most efficient simulated TC. (**b**) A diagram of a typical simulated scenario belonging to the double-stage OTC at 0.1% of prevalence. The header gives basic information about the population size used in simulation, prevalence, number of reactions used in total and basic statistics. Each rectangle represents one RT-qPCR reaction, which can be marked as negative group (blue) positive group (yellow) or positive individual (red). The lines connect the reactions that are subsequent performed in this scenario (i.e. stages). All rectangles contain four rows of text, which show from top to bottom: (1) stage of the testing configuration, (2) number of individuals in the group, (3) percent of all reactions that belong to that reaction type (e.g. first group of the second stage that is negative) and (4) percent of the tested population that was submitted to that reaction type.

We found that two-stage OTCs can boost spreader detection in 15.38 times in the lowest prevalence of 0.1% (Fig. 3a), but become less efficient as prevalence increases, and in the higher prevalence of 30% it was no better than individual testing. Although positive groups need an individual test in the second stage, mainly in low prevalence, 97.5% of the population was part of negative groups and therefore will request only one stage to obtain the result (Fig. 3b). Although the figure reflects only one simulation scenario, the sensitivity was equal to 100% in all double-stage iterations tested at 0.1% of prevalence. It is important to mention that for group sensitivity, showed at the Fig. 2a, we considered sensitivity as the percent of samples with detectable Ct values in N or RdRp genes. Otherwise, if only the E gene had a detectable Ct value, it was considered negative. However, during the simulation of testing configurations just one detectable gene was enough to be considered as a positive group (i.e. E, N or RdRp). Thus, the sensitivity of a testing configuration starting with a group may be higher than a group with the same size. Therefore, exclusively for the simulation of testing configurations, this rule considering N or RdRp genes was only applied when individual testing was reached (i.e. dilution effect is nonexistent) to improve the general sensitivity of the testing configuration that is under test whereas the final individual result properly considers only SARS-CoV-2 specific genes.

### Multi-stage group testing can pinpoint more spreaders and mitigate dissemination

**Figure 4:**
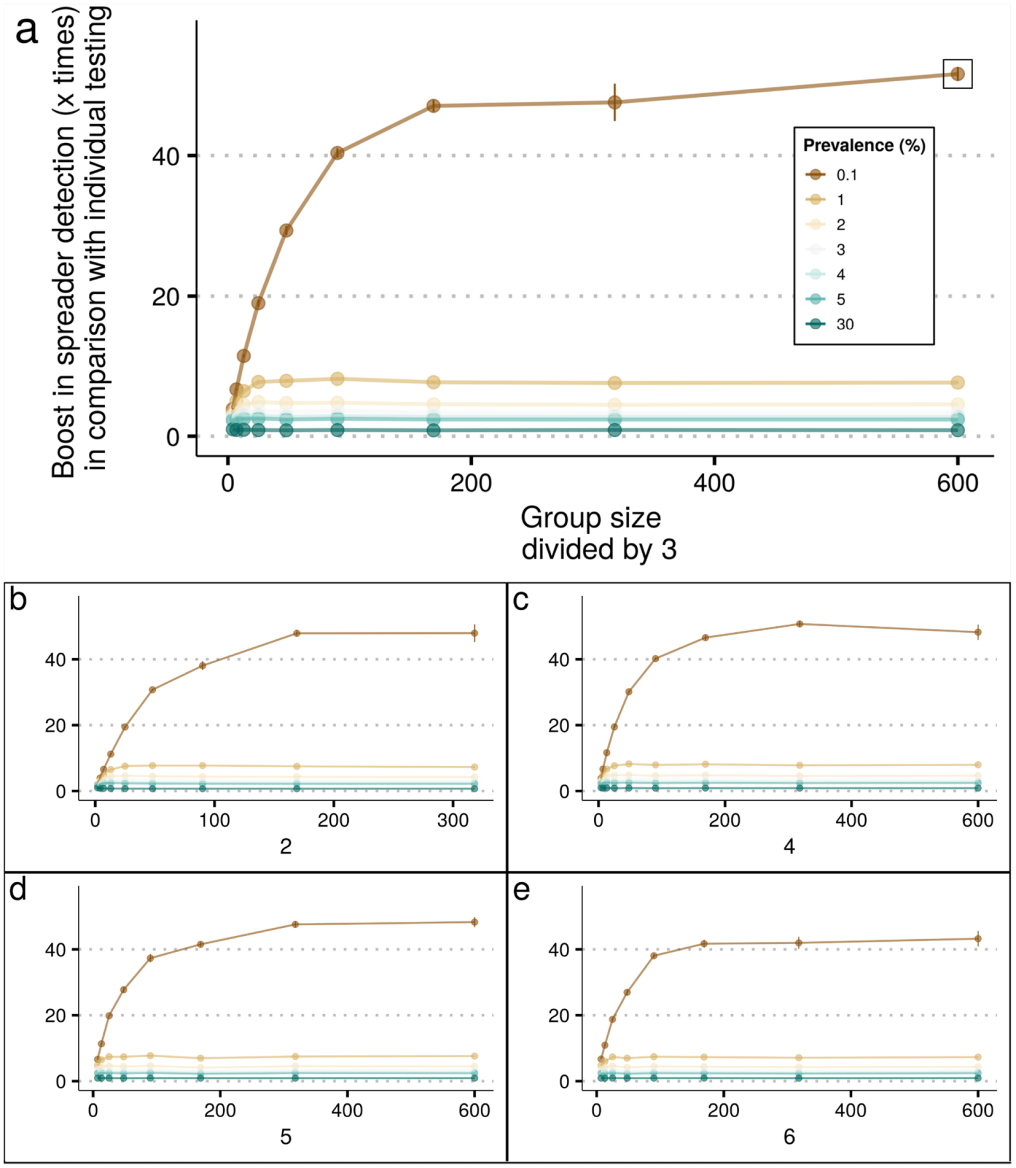
The boost in screening power by the use of multi-stage testing configurations. Each plot shows the boost among different denominators (i.e. the way the master group is divided). (**a**) The denominator with the multi-stage OTC for 0.1% of prevalence for which the master group is subsequently divided by three. The OTC is highlighted by a square around the point. (**b**) Master group divided by two. (**c**) Master group divided by four. (**d**) Master group divided by five. (**e**) Master group divided by six.

Multi-stage configurations may require more time than two-stage configurations to pinpoint spreaders, from sample collection to result, as more subsequent tests may be necessary for positive groups. On the other hand, performing several stages could save more reagents than two stages only and therefore should be considered^33^. To investigate the *boost in spreader detection* when several stages are allowed, we used our *poolingr* package to simulated testing configurations with their group sizes subsequently divided by values ranging from 2 to 6 , which generated configurations with a variable number of stages, varying from 2 to 7 . We found that multi-stage are usually more efficient than two-stage configurations to boost spreader detection. Multi-stage OTCs have relatively larger initial group sizes and lower sensitivity, considering a disease prevalence that is up to 5%. As disease prevalence increases, the master group size in the OTC decreases until testing configuration is not useful anymore at 30% of prevalence. Thus, multi-stage OTCs are expected to be more efficient in a lower disease prevalence. However, as the master group size in prevalence-specific multi-stage OTCs increase, the number of stages that are necessary to reach individual testing increases along. For the lowest tested prevalence of 0.1%, more than six hundred individuals can compose the master group to save more tests even considering a notable dilution effect in such large groups (Fig. 4, OTC had group size of 600 , divided by three with general mean sensitivity of 90.55% , i.e. observed/expected spreader ratio). In the best multi-stage OTC detected, the simulated scenario of ten thousand individuals, that was closer to all ten iterations average, identified 9 spreaders using 173 RT-qPCR reactions. If the same number of reactions are used to test individuals randomly, it would otherwise probably miss all 10 spreaders in the population as mathematically a total of 0.173 spreaders would be identified (i.e. 0.1% of 173). Therefore, the multi-stage OTC at 0.1% of prevalence, is predicted to identify 51.61 times more spreaders than individual testing (i.e. *boost in spreader detection*). Although multi-stage OTCs are in general more useful at a lower disease prevalence, the multi-stage OTCs were more efficient than individual testing for prevalence values up to 5% . However, it is important to express that all prevalence values between 5 and 30% were not simulated in this study, therefore the real threshold is unknown.

**Figure 5:**
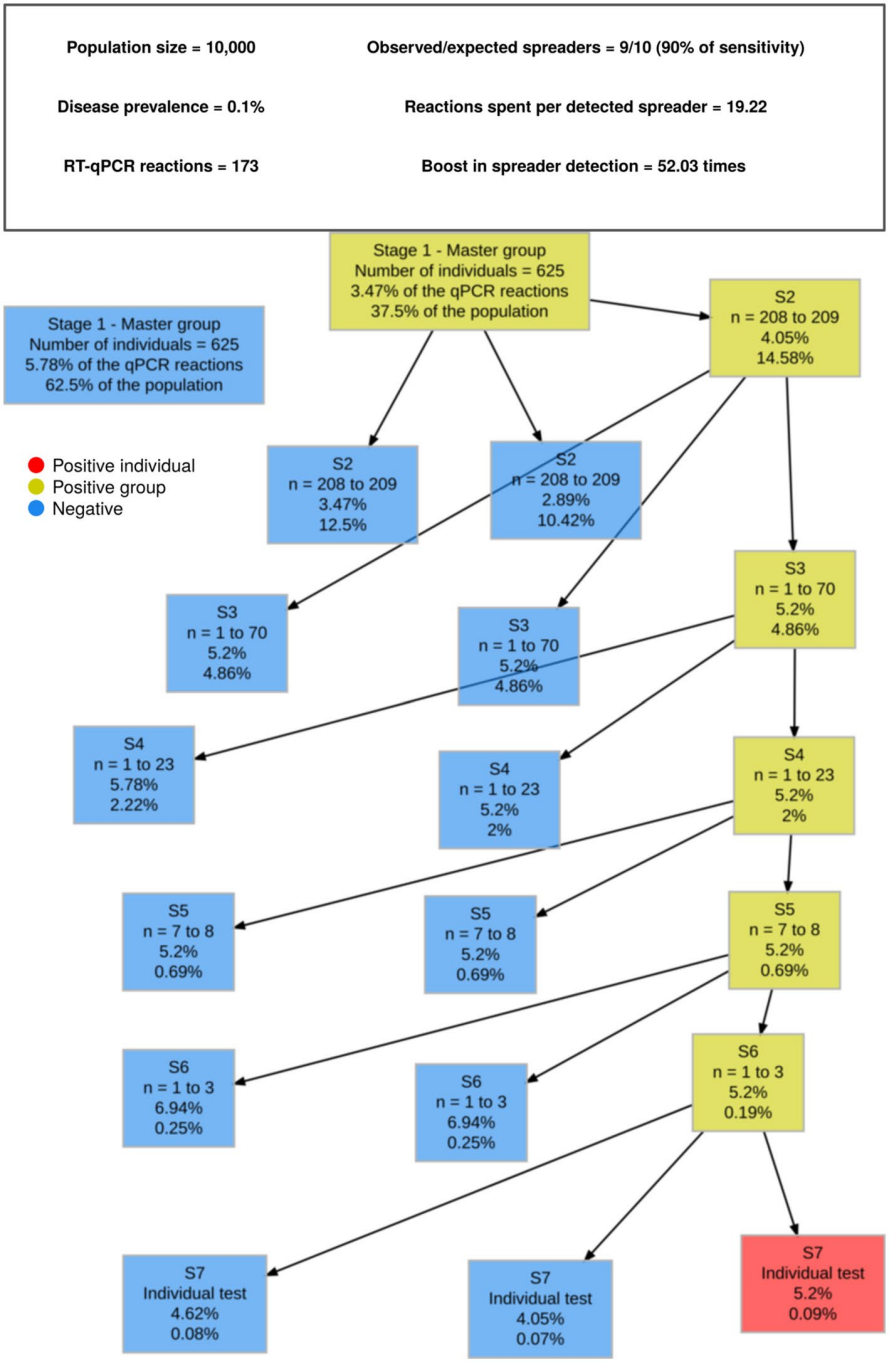
A diagram of a simulated scenario belonging to the multi-stage OTC at 0.1% of prevalence, which has the *boost in spreader detection* closest to all iterations average. The header gives basic information about the population size used in simulation, prevalence, number of reactions used in total and basic statistics. The majority of the population (i.e. 62.5% ) will retrieve as negative in the first stage (i.e. master group), 85.42% in second and progressively smaller percentages in subsequent stages. This testing configuration will require seven states to reach individual testing in a spreader.

The multi-stage OTC at 0.1% of prevalence (i.e. 323 subsequently divided by three ) includes the master group followed by six additional stages in total. In this OTC, the first stage (i.e. master group) generated 62.5% of the results, as the majority of the master groups are negative (Fig. 5). Thus, even considering several stages, most of the results may be obtained in large master groups that test negative. On the other hand, positive individuals will be unknown until individual testing is accomplished in the seventh stage. This simulated scenario obtained a general sensitivity of 90%, which is close to the mean of scenario iterations (0.905% with a 0.097% standard deviation). In this simulated scenario, more than 85.42% of the tested individuals had a negative result after the second OTC stage. Thus, even if a laboratory setup is able to perform only one stage per day, only 14.58% of the tested individuals would be requested to stay on preventive quarantine 48 hours after the test and less than 5% after 72 hours.

## Discussion

The world will probably face new pandemics more frequently from now on, which will require a stronger and more integrated surveillance for emerging diseases. The current state of deforestation, expansion of agriculture and exploitation of wild species will increase the risk of human contact with new pathogens underlying zoonoses. In fact, the majority of emerging infectious diseases are zoonoses and most of them originate in wildlife^34^. As for a new zoonosis the severity of the disease and human-to-human transmissibility is unknown, new diseases such as COVID-19 can become a severe new pandemic rather quickly. Thus, once a pathogen has the genetic code reported, it is important to consider the implementation of testing programs. If a testing program targets the best use of a limited amount of resources, identifying as many potential spreaders as possible may be the most efficient way to contain the dissemination. This is expected because spreaders that are identified and consequently informed about their condition, have a higher chance to voluntarily quarantine and then decrease the basic reproduction ratio (${R}_{0}$) of a pathogen. Group testing can be an alternative to achieve such a target, but low number of stages in some hierarchical OTCs may be a limiting factor to significantly increase the efficiency in spreader detection.

Simulation of multi-stage OTCs could assist in the prediction of more efficient testing strategies that would consequently save and optimize reagent use in the current COVID-19 pandemic or at any new to come. Moreover, the use of viral loads, such as the populational real RT-qPCR Ct values, can assist in the search for a suitable strategy based on the expected dilution effect caused by grouping and prevalence when implementing a specific test type. Information on the viral load may be crucial to better understand the effect of certain grouping strategies, mainly on the samples that have lower viral loads (i.e. higher Ct values). Thus, larger groups can more frequently miss individuals with relatively lower viral load, which may be undetected spreaders. On the other hand, although the association between viral load and infectivity among our samples in unknown, individuals with lower viral loads may be less effective spreaders^37^ and therefore contribute less to the ${R}_{0}$.

Although multi-stage OTCs can save reagents, it can take longer to retrieve the results and delay time-sensitive actions for dissemination control such as the isolation of infected individuals before they spread a disease (i.e. quarantine period). However, by setting a maximum number of days to achieve a diagnostic in *poolingr* it is possible to select the best strategy that can be performed within a predefined time-frame. Strategies allowing several stages are more feasible in testing programs that are able to run more than one subsequent test assay in the same day because the result of a previous stage needs to be considered to plan the next associated stage. In a fast-spreading pandemic such as COVID-19, it is essential to identify spreaders as quick as possible to increase the quarantine ratio among possible spreaders. In spite of this, even when it is not possible to run several stages in a short amount of time, multi-stage OTCs can still be useful to increase the number of spreaders in quarantine. For example, if a testing program recommends quarantine to the individuals that belong to positive groups, it would invariably lead to an increase in the number of spreaders that are isolated. Although it would require some negative individuals to needless quarantine, at least until their individual results are released, this procedure could significantly decrease ${R}_{0}$. Consequently, a curfew or lockdown due to uncontrolled disease dissemination become less likely to be implemented. However, as multi-stage OTCs can delay positive results, for patients with symptoms or with a scheduled medical procedures an individual test may be the best option.

The automation of multi-stage OTCs on the bench can be laborious. If a pipetting robot is nonexistent, the reallocation of individuals from positive groups on subsequent assay runs may require too much time and attention from a lab technician, ultimately increasing the errors due to pipetting. A possible solution could be a software to assist lab technicians on the plate design of runs that depend on the results of a previous stage, given the assumptions of the OTC under use. Moreover, due to the dilution, OTCs with large initial groups tend to display lower sensitivity in comparison with strategies that make use of smaller master groups. If a testing program implements such OTCs, it should be targeting more power to pinpoint infected individuals in a population (i.e. *boost in spreader detection*) instead a good sensitivity in individual results. Moreover, the dilution effect might be different when RNA extraction is performed using groups instead individuals. Here we assume that individually RNA extractions were grouped as a configurations requires it. Thus, the sensitivity of testing configurations using large groups might have lower *boost in spreader detection* when extraction is accomplished directly from groups. Moreover, distribution of target nucleic acid in the patient sample is not always homogeneous. Thus, the use of RNA extraction from groups could decrease the sensitivity of a group in a way that is not considered by the uniform dilution effect implemented by our simulations.

Simulation of grouping scenarios, as proposed in this study, is considerably more compute-intensive when compared with previously described statistical models for objective functions used to estimated OTCs. Whereas previous models can determine the OTC in a couple of minutes or seconds, each simulated scenario may be computed separately in our current algorithm. Therefore, an analysis with a fair number of scenarios may take a long *in silico* run, even on multiple cores. On the other hand, simulating scenarios may be a more flexible way to test the effect of variables, such as the dilution effect caused by the group size (i.e. decrease of sensitivity as group size increases). Moreover, by screening the results of simulations it is possible to visualize the expected variation among permutations. One strategy to decrease the computer power needed to obtain an OTC from simulations could be the implementation of machine learning strategies with preexisting data, allowing more rapid prediction of OTCs for different scenarios (e.g. Ct values and prevalence). Another possible limitation is that we explored the dilution effect of the group size whereas the sensitivity and specificity of the test assay itself was set to 100% (i.e. $assay.sensitivity$ and $assay.specificity$). However, this may not be the case in actual laboratory setups. Thus, if these values are known, it is advisable to run simulations considering them as parameters in to make the simulation more reliable. Although simulation can be demanding, existent objective functions deal with the dilution effect by setting *I*, which delimits the maximum group size allowed in a specific OTC^26^. Thus, a laboratory that intends to use group testing based on such functions must set a threshold for max group size beforehand. However, *I* may not be trivial to calculate. For example, initially setting *I* makes it impossible to quantify how the dilution effect will shift the general test sensitivity as group size increases (Fig. 2). Thus, the use of *I* can generate biased OTCs as the sensitivity is considered erroneously constant among different group sizes.

Currently, *poolingr* package allows the simulation of hierarchical OTCs. A possible future improvement could be to include simulation of the matrix and cube^35^ grouping strategies. Such improvement would allow potentially more efficient OTCs and ultimately more complex combinations of originally unrelated strategies such as hierarchical followed by a cube method. Therefore, more complex testing programs that are able to allocate up several stages could considerably increase their power to identify spreaders by employing multi-stage group testing. We present an open-source package to simulate testing configurations, which we used with observed SARS-CoV-2 viral loads. Our results indicate that multi-stage testing configurations can enhance the power to identify spreaders and consequently mitigate disease dissemination. Thus, we expect this study to contribute with present and future efforts to fight against emerging diseases throughout well-planned mass testing programs.

## Methods

### Ethics

The use of clinical swab samples analyzed at Functional Genomics Center (BIOCEMA) was approved by the Ethics Committee on Human Research (ESALQ/USP, protocol number CAAE 34308620.0.0000.5395). The requirement for informed consent was waived by the committee as this study was part of a public health surveillance and outbreak investigation in Brazil. Therefore, this study was performed in accordance with the relevant Brazilian laws and regulations.

### Clinical specimens and RNA extraction

Nasopharyngeal swab samples were collected by healthcare technicians and placed in 15mL-conical sterile tubes with 2 mL of saline solution and maintained at $4\,^{\circ }$C. All specimens were aliquoted in 2 mL tubes and individual nucleic acid extraction was conducted ed upon arrival in the laboratory with the MagMAX$^{\mathrm{TM}}$ Viral/Pathogen Nucleic Acid Isolation Kit, (Applied Biosystems, cat number) in KingFisher equipment (Thermo Scientific, USA). Two hundred microliters of each sample were used in RNA extraction. The remainder was stocked at $-80\,^{\circ }$C.

### Real-time RT-qPCR

Samples were tested according to the Charitè protocol (E gene, N gene, and RdRp^36^) with the GeneFinder COVID-19 PLUS RealAmp kit (OSANG Healthcare, Korea). The human RPP30 (RP) gene was used as endogenous control. Procedures and thermocycler conditions were conducted according to manufacturer instructions on Quantstudio 6 or 12k Flex machines (Applied Biosystems, CA, US). Only individuals with Ct below 40 in at least one specific primer-probes sets (N gene or RdRp) for SARS-CoV-2 were carried to the in silico simulations. All runs were validated by vector positive control and water as a negative control.

### Statistics

The objective function proposed in this study is based on the simulation of grouping scenarios, under certain testing configurations (e.g. different group size and prevalence), which are ranked by a *boost in spreader detection*. It selects samples from a binary index with randomly placed ‘0’ and ‘1’ values representing healthy and diseased individuals, respectively. As the index represents a population, the 0/1 rate is defined by the disease prevalence. If a positive individual is present in a group (i.e. index chunk), a set of individual Ct values observed in vitro is randomly assigned to represent this individual in simulation. Thus, depending of the Ct expected for the group, in which the individual is placed, the individual may be missed or detected (i.e. above or below the Ct detection limit). The calculation of the group Ct can be performed by the function CtInPool, which estimates log-linear Ct increase along target nucleic acid quantities. However, as groups can have more than one positive individual, all individual theoretical relative nucleic acid quantities (i.e. $\frac{1}{(pcr.efficiency+1)^{ct.sample}}$) were summed up and divided by the group size (i.e. *group*.*rna*.*quantity*). Therefore, a two-step procedure using the uniroot function from stats R package^38^ was used to estimate the Ct value for groups. In the first step uniroot resolves the equation $\frac{group.rna.quantity-1}{x}$, where *x* is the unknown *dilution*.*index* of the group. In the second step, the Ct for the pool was estimated by the function $(pcr.efficiency+1)^{x}-dilution.index$, where *x* represents the group Ct. This two-step procedure was implemented in the internal *poolingr* createClusterInfo function. A scenario is defined by (1) population index, (2) initial group size and (3) a denominator. The denominator will define how the master group with be split in the subsequent stages by $\frac{group size}{denominator}$, if the simulated group holds at least one positive sample. The algorithm was implemented in the R package *poolingr*, which is freely available on https://bitbucket.org/viniciushdasilva/poolingr.

We simulated testing configurations with group sizes ranging from 2 to 600, which split subsequently by $denominators$ from 2 to 6. For example, a $denominator$ equal to two will result in a split of groups by half subsequently after each stage. In the *poolingr* package, $denominator$ and all other parameters were defined in the simulateTC function. After the *poolingr* installation is complete, all the results presented in this manuscript can be reproduced by the examples included in the vignette, which can be accessed with vignette(“poolingr”).

## Data Availability

The data supporting all findings of this study are available within the article and in the reproducible example in the *poolingr* package repository. The raw quantitative PCR data is subjected to personal data protection.
